# Clinical outcomes of genomically guided trametinib monotherapy across cancer types: results from the IMPRESS-Norway trial

**DOI:** 10.2340/1651-226X.2026.45086

**Published:** 2026-02-10

**Authors:** Kathinka Schmidt Slørdahl, Katarina Puco, Ragnhild Sørum Falk, Ingrid Dyvik, Sigmund Brabrand, Pitt Niehusmann, Eli Sihn Samdal Steinskog, Egil S. Blix, Åsmund Flobak, Irja Alida Oppedal, Sebastian Meltzer, Cecilie Fredvik Torkildsen, Hanne Blakstad, Kristina Lindemann, Anita Amundsen, Sigbjørn Smeland, Kjetil Taskén, Åslaug Helland, InPreD Consortium

**Affiliations:** a Department of Oncology, Oslo University Hospital, Oslo, Norway; b Institute for Cancer Research, Oslo University Hospital, Oslo, Norway; c Oslo Centre for Biostatistics and Epidemiology, Oslo University Hospital, Oslo, Norway; d Institute of Clinical Medicine, Faculty of Medicine, University of Oslo, Oslo, Norway; e Department of Pathology, Oslo University Hospital, Oslo, Norway; f Department of Oncology and Medical Physics, Haukeland University Hospital, Bergen, Norway; g Department of Oncology, University Hospital of North Norway, Tromsø, Norway; h The Cancer Clinic, St. Olavs University Hospital, Trondheim, Norway; i Department of Clinical and Molecular Medicine, Norwegian University of Science and Technology, Trondheim, Norway; j Department of Biotechnology and Nanomedicine, Sintef Industry, Trondheim, Norway; k Department of Thoracic Medicine, Haukeland University Hospital, Bergen, Norway; l Department of Oncology, Akershus University Hospital, Lørenskog, Norway; m Department of Gynecology and Obstetrics, Stavanger University Hospital, Stavanger, Norway; n Centre for Cancer Biomarkers, University of Bergen, Bergen, Norway; p Section for Gynecological Oncology, Department of Surgical Oncology, Oslo University, Oslo, Norway

**Keywords:** Precision medicine, biomarkers, trametinib, uveal melanoma, Norway

## Abstract

**Background and purpose:**

Molecular profiling guides cancer treatment, by identifying actionable genomic alterations. The IMPRESS-Norway trial (NCT04817956) is a nation-wide precision medicine trial evaluating the efficacy of approved cancer drugs on a novel indication in patients with advanced cancers harbouring potentially actionable alterations. Trametinib, a selective MEK1/2 inhibitor targeting the Mitogen-Activated Protein Kinase (MAPK) signalling pathway, is approved for BRAF V600 mutant melanoma but may also show activity in tumours with other alterations. This sub-study aimed to assess the efficacy of trametinib monotherapy across tumour types with alterations activating the MAPK signalling pathway.

**Patient/material and methods:**

In the IMPRESS-Norway trial patients are screened with the TruSight Oncology 500 panel or circulating tumour DNA profiling. Eligible patients are offered biomarker matched targeted therapies. In this subgroup analysis, we identified patients treated with trametinib monotherapy. Primary endpoints were disease control rate (DCR) after 16 weeks and safety. Secondary endpoints included progression-free survival (PFS) and overall survival (OS).

**Results:**

DCR after 16 weeks of treatment was 39% in 52 response evaluable patients, with four patients (8%) experiencing partial response, and 16 (31%) stable disease. Responses were seen in tumours harbouring BRAF fusions, GNA11, GNAQ, KRAS, NF1, and NRAS alterations, most frequently in low-grade serous ovarian cancer, central nervous system tumours, and uveal melanoma. Forty-eight percent of patients experienced treatment-related adverse events, including two treatment related deaths. Median PFS and OS were 4 and 9 months, respectively.

**Interpretation:**

Trametinib monotherapy achieved a 39% DCR in patients lacking standard options, supporting further studies to confirm efficacy and identify predictive biomarkers for treatment response.

## Introduction

Molecular profiling has become an important part of cancer diagnostics in several cancer types, guiding treatment selection, and identifying emerging therapeutic targets or resistance mechanisms at progression. Additionally, the European Society for Medical Oncology (ESMO) Precision Medicine Working Group recommends multigene sequencing to identify patients eligible for clinical trials [1].

The IMPRESS-Norway study is an ongoing prospective, non-randomised clinical trial evaluating efficacy of off-label, commercially available anti-cancer drugs prescribed for patients with advanced cancer diagnosed with potentially actionable alterations revealed by molecular profiling [2]. Preliminary results have shown an overall disease control rate (DCR) of 40% [3].

One of the available drugs in the IMPRESS-Norway study is trametinib. Trametinib is a reversible and selective inhibitor of MEK1 and MEK2 kinases resulting in MAPK signalling pathway inhibition [4]. Trametinib received regulatory approval for unresectable/metastatic melanoma based on the Phase III METRIC trial, which demonstrated significantly improved progression-free survival (PFS) and overall survival (OS) compared with chemotherapy in patients with BRAF V600E/K mutant metastatic melanoma [5]. Trametinib has later become standard treatment in combination with dabrafenib for several tumour types harbouring the BRAF V600 mutations.

Hyperactivation of the MAPK signalling pathway, by activating mutations and fusions of protein kinases and inactivation of tumour suppressor genes, is promoting cell proliferation and tumour growth. Therefore, it is reasonable to assume that trametinib could be effective in the presence of MAPK pathway activating alterations beyond BRAF V600. Earlier studies have shown that trametinib may be effective in tumours harbouring GNAQ, NF1, KRAS, or NRAS alterations, supporting the rationale to investigate the efficacy of trametinib monotherapy [6–12].

This subgroup analysis aimed to evaluate the efficacy of trametinib monotherapy in patients with alterations in the MAPK signalling pathway enrolled in the IMPRESS-Norway trial.

## Patients/material and methods

### Study design

IMPRESS-Norway is a prospective, non-randomised, nationwide clinical trial evaluating the efficacy of off-label, commercially available and approved anti-cancer drugs in patients with advanced cancers harbouring potentially actionable genomic alterations. The study employs a combined umbrella and basket design with a Simon two-stage model to assess potentially effective biomarker-drug combinations for specific indications [2].

All patients were screened using the comprehensive genomic profiling panel TruSight Oncology 500 (TSO500, Illumina). For patients with no available tissue, genomic profiling of circulating tumour DNA (ctDNA) using the FoundationOne Liquid CDx assay (Foundation Medicine, Inc.) was performed. A predefined biomarker list was used to allocate patients to treatment with trametinib, and the following potentially actionable alterations were included: BRAF activating fusions, activating mutations in GNAQ, GNA11, GNAS, MAP2K1 or MAP2K2, NF1 mono- or biallelic inactivation (CNS tumours only), NRAS activating mutations or amplification, HRAS and KRAS activating mutations (low grade serous ovarian carcinoma only), and MAP2K4, MAP3K1 or LZTR1 biallelic inactivation. MAP2K1, MAP2K2, MAP2K4, MAP3K1 and LZTR1 were abandoned during the study due to slow accrual and uncertain predictive value of the biomarkers. As of October 2025, trametinib was no longer available in the study.

### Study population

Adult patients with advanced solid or haematological malignancies who had progressed on all standard therapies were eligible for inclusion. DNA/RNA profiling had to reveal one of the pre-defined molecular biomarkers for trametinib treatment, adequate performance status and organ function, a life expectancy of at least 3 months, and meet all study and drug-specific inclusion and exclusion criteria.

Patients included in the response-evaluable population used for efficacy analysis had received at least one cycle of trametinib (28 days) and had been evaluated according to protocol. Clinical deterioration and inability to complete per protocol evaluation were considered to be signs of progressive disease at the discretion of the treating physician. All treated patients were included in the safety analysis.

Patients included in this subgroup analysis were enrolled from April 2021 and followed up until data cut-off at September 9, 2025.

### Study endpoints

The primary study endpoints were DCR after 16 weeks of treatment and treatment safety. DCR was defined as radiologically confirmed complete response (CR), partial response (PR), or stable disease (SD), after a minimum of 4 weeks. RECIST v1.1 [13] and RANO [14, 15] evaluation criteria were used for solid cancers and primary brain tumours, respectively, while ELN-AML criteria [16] were used for haematological cancers. Patients were evaluated at treatment weeks 8, 16, 24, and every 3 months thereafter.

Toxicity was assessed by the Common Terminology Criteria for Adverse Events v5.0. Treatment-related adverse events (TRAEs) ≥ grade 3 and treatment related serious adverse events of any grade were collected up to 30 days after last treatment dose.

Secondary endpoints were PFS and OS.

### Treatment with trametinib

Patients started at a dose level of 2 mg once daily. In cases of toxicity the dose was reduced according to protocol. Left ventricular ejection fraction was evaluated in all patients at baseline, after 1 month and every 3 months thereafter. Patients received treatment until disease progression, unacceptable toxicity, death or withdrawal of any reason. However, treatment beyond progression was permitted in certain cases.

### Data collection and statistical analysis

Data included in this analysis were collected from the electronic case report form Viedoc. Stata version 18 was used for statistical analysis. Patient characteristics and tumour responses were summarised using descriptive statistics. DCR was calculated as the proportion of patients with CR, PR, or SD in the response evaluable population. PFS was defined as the time from treatment initiation to the first recorded progression or death of any cause. Event date for progression was defined by the date of CT- or MRI-scan or the date of bone marrow/blood procedure for haematological cancers. Otherwise, in cases of clinical deterioration, the time point of progression was the visit date where the unequivocal progression was recorded. Patients who stopped treatment due to any cause without recorded progression or death were censored for PFS time at the date of their last visit. OS was defined as the time from treatment initiation to death of any cause, or censored at last date known to be alive, whichever occurred first. PFS and OS were assessed by the Kaplan–Meier method and presented with accompanying 95% confidence intervals (CI).

## Results

### Patient and tumour characteristics

In total, 65 patients started treatment with trametinib monotherapy. Median age at treatment start was 62 years (range 19–83), and 63% of treated patients were females. The majority of patients had an ECOG performance status of 0–1 (81%) and had received 1–3 prior treatment lines. The most common included tumour types were uveal melanoma and ovarian cancer. Patient characteristics are presented in Table 1.

**Table 1:** Characteristics of patients treated with trametinib monotherapy, *n* = 65.

Characteristics	*n* (%)
Age, years, median (min–max)	62 (19–83)
Sex	
Female	41 (63)
Male	24 (37)
ECOG performance status	
0	23 (35)
1	30 (46)
2	12 (19)
Tumour types	
Ovarian cancer	14 (22)
Uveal melanoma	14 (22)
CNS tumour	10 (16)
Colorectal cancer	7 (11)
Non-small lung cancer	4 (6)
Haematological malignancy	4 (6)
Cholangiocarcinoma	3 (5)
Neuroendocrine carcinoma	2 (3)
Neuroendocrine tumour	2 (3)
Prostate cancer	2 (3)
Mucosal melanoma	1 (1)
Pancreatic cancer	1 (1)
Uterus cancer	1 (1)
Previous treatment lines	
None (radiation/surgery only)	2 (3)
1 line	13 (20)
2 lines	29 (45)
3 lines	10 (16)
4 lines	4 (6)
5 lines	7 (10)

The most frequently detected actionable alterations among patients who received trametinib were NRAS Q61 H/K/L/R (*n* = 14), and KRAS G12 D/V (*n* = 9) identified in different tumour types, GNAQ Q209 P/L (*n* = 8) identified in uveal melanomas, and NF1 inactivating mutations (*n* = 8) identified in central nervous system (CNS) tumours. Detailed overview of actionable alterations in treated patients is presented in Supplementary Table 1.

The median time from inclusion to the data-cutoff was 22 months (range 0.4–35).

### Efficacy assessment

**Figure 1:**
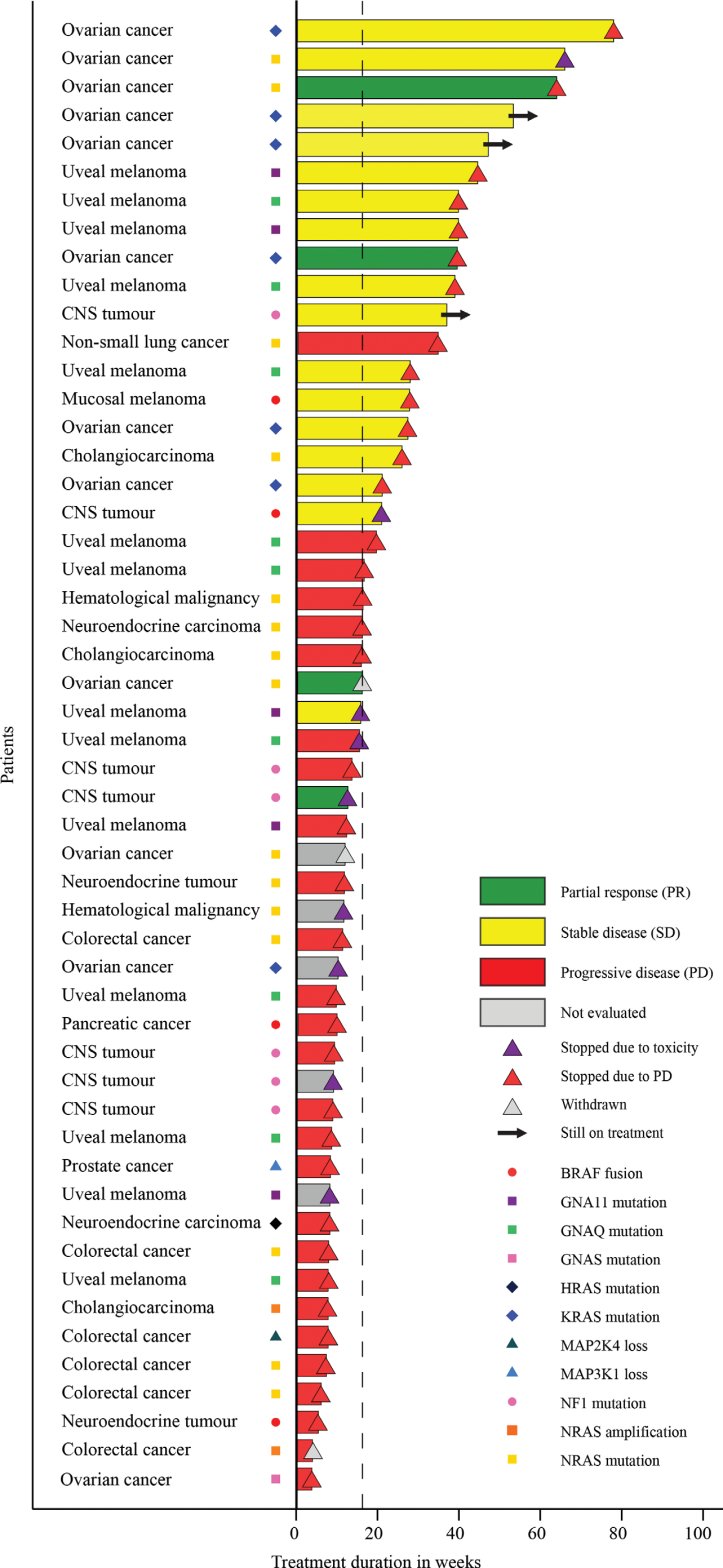
Swimmers plot of ‘response evaluable’ patients treated with trametinib monotherapy, *n* = 52.

Of the 65 patients who started treatment, 13 were considered not evaluable according to protocol. Among the 52 patients in the response evaluable population, the DCR was 39%; four patients (8%) achieving PR and 16 (31%) SD after 16 weeks of treatment (Figure 1). No CR were observed. Three patients with low-grade serous ovarian cancer (LGSOC) and one with high-grade astrocytoma with piloid features experienced PR. In addition, six patients with LGOSC, one patient with recurrent pilocytic astrocytoma and one patient with diffuse leptomeningeal glioneuronal tumour, six patients with uveal melanoma, one patient with mucosal melanoma and one patient with cholangiocarcinoma had SD. Three of these patients are still on treatment. Three patients received treatment beyond progression.

PR and SD were observed in tumours harbouring BRAF fusions, and GNA11, GNAQ, KRAS, NF1 and NRAS activating or inactivating alterations, whereas no responses were seen in patients with tumours harbouring NRAS amplifications, HRAS activating mutations or MAP2K4 and MAP3K1 biallelic inactivation. A detailed overview over responses according to biomarkers and tumour types are shown in Supplementary Table 1.

**Figure 2:**
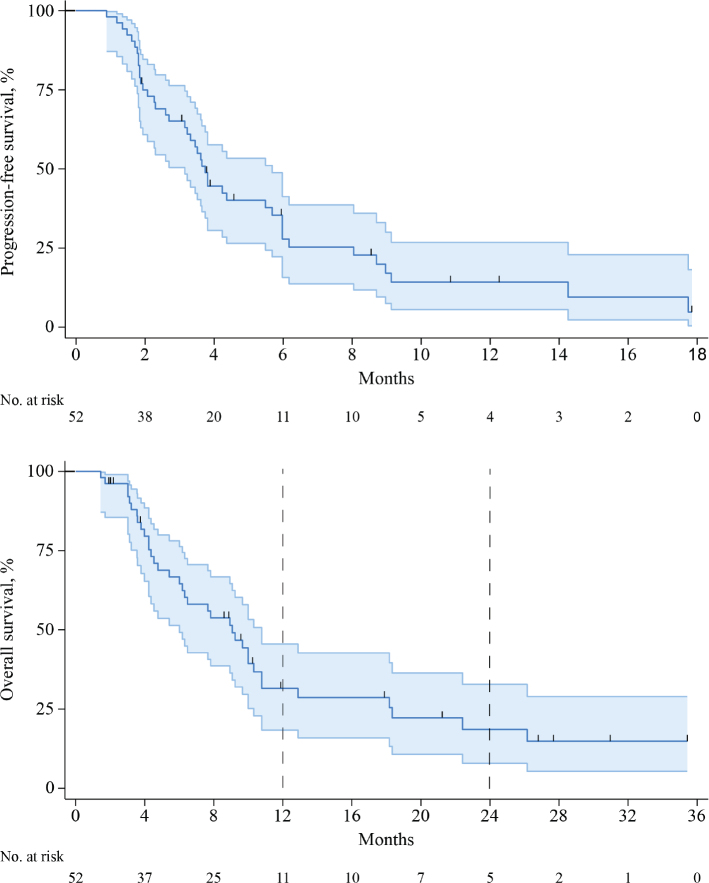
Progression-free survival and overall survival (with 95% CI) of ‘response evaluable’ patients treated with trametinib monotherapy, *n* = 52. (A) Progression-free survival, (B) Overall survival.

In the response evaluable population, the median OS was 9 months (95% confidence interval [CI]: 6–10), median PFS was 4 months (95% CI: 3–6) and 1-year PFS was 14% (95% CI: 6–27) (Figure 2).

### Safety

Out of 65 patients treated with trametinib, 31 (48%) experienced TRAEs. In total, 58 TRAEs were reported, with 38 (66%) being serious adverse reactions. The most commonly reported TRAEs were rash, increased liver enzymes, and mucositis. Six suspected unexpected serious adverse reactions (SUSARs) were registered, including myocardial infarction, sepsis, stroke, and thromboembolic event all registered in one patient, and an additional two thromboembolic events in two patients. Two treatment-related deaths were reported, one related to a thromboembolic event, and the other to an intra-abdominal haemorrhage. A total of 14 patients (22%) stopped treatment due to TRAEs. The overview of all reported TRAEs is shown in Supplementary Table 2.

## Discussion and conclusion

This subgroup analysis of clinical outcomes in patients treated with trametinib monotherapy in the IMPRESS-Norway trial, demonstrated clinical benefit with a DCR of 39%. The observed responses were seen in patients with LGSOC, CNS tumours, uveal and mucosal melanoma and cholangiocarcinoma harbouring BRAF fusions, and GNA11, GNAQ, KRAS, NF1 and NRAS activating or inactivating mutations.

Patients with LGSOC represented the largest subgroup with observed clinical benefit, including both PR and SD. Trametinib monotherapy has been shown to be effective in this patient group in a recurrent setting, regardless of mutational status, although the effect seems to be stronger in those with BRAF/NRAS/KRAS mutations [9]. According to the ESMO guidelines, patients with LGSOC relapse should be considered for treatment with trametinib after platinum failure [17]. This study supports previous findings that this patient group may benefit from trametinib monotherapy. A more detailed analysis of this cohort will be presented in a separate publication.

Patients with metastatic uveal melanoma have limited systemic treatment options. Mutations in GNA11 and GNAQ, which occur in 80–90% of tumours, represent potential molecular targets [18]. A systematic review by Steeb et al. [19] summarises the literature on MEK inhibitors in uveal melanoma. Selumetinib (MEK1/MEK2 inhibitor) was described being the best documented substance, where an effect on PFS was found in a phase II trial [6], but not confirmed in the subsequent phase III- trial [20]. Previous studies on MEK inhibitors have shown only low response rates; however, trametinib remains less well studied [19]. In this study no patients had PR/CR, but several had SD. Metastatic uveal melanoma can follow an indolent course; however, all patients were required to have disease progression before treatment start. Therefore, while part of the disease stabilisation may be due to the indolent nature of metastatic uveal melanoma, a treatment effect cannot be ruled out. The small patient population should also be considered. Thus, these results should be interpreted with caution.

The EANO guidelines recommend testing of NF1 in gliomas, glioneuronal, and neuronal tumours due to the potential efficacy of MEK inhibitors in these patients [21]. This effect was demonstrated in this study, showing both PR and SD in these patients. A more detailed analysis of the CNS patients will be presented in a separate publication.

The overall safety profile of trametinib was, in general, as expected [22]. However, two treatment-related deaths were reported, including one patient with advanced age, and multiple comorbidities, experiencing severe toxicity not previously reported. Out of six reported SUSARs, three were thromboembolic events, known side effect of trametinib, but still needed to be reported due to regulatory demands. The relatively high treatment toxicity must therefore be balanced against the anticipated benefit, particularly in patients with advanced age and comorbidities.

A limitation of this study is the small number of patients in each biomarker and tumour type. Another limitation is that the two largest groups of patients with effect have tumour biology in which the tumour progression may be slow, potentially influencing the primary endpoint. The strengths of this study include the prospective design and the opportunity to treat and compare results from different types of cancer based on the same molecular target. Furthermore, this trial is conducted in a near real-world setting where heavily pretreated patients with ECOG 0–2 and comorbidities received experimental treatment.

## Conclusion

Patients treated with trametinib monotherapy in this biomarker driven study achieved a DCR of 39%, representing a clinically meaningful result in a patient population with no standard treatment options available. However, toxicity profile may be challenging in patients with poor ECOG status and comorbidities. Given these findings, further research is needed to confirm whether this effect persists in a larger cohort and to investigate potential molecular or other predictive factors that may forecast treatment response.

## Supplementary Material



## Data Availability

The full clinical dataset consists of de-identified patient-level data obtained from VieDoc. The sponsor and data owner is Oslo University Hospital. Access to full raw patient-level data is limited, but project partners can apply for access through the data and biobank committee of the trial, in accordance with Data Privacy and Ethical Approval for the study project. All authors have full access to complete study data, study analysis performed, tables and figures. The study protocol is available.
