# Supplementary material for: Clinical outcomes of genomically guided trametinib monotherapy across cancer types: results from the IMPRESS-Norway trial
Source: Acta Oncol. 2026 Feb 10;65:45086. doi: 10.2340/1651-226X.2026.45086 (PMC12902912; doi:10.2340/1651-226X.2026.45086)
Supplement: Supplementary file 1 [file AO-65-45086-s1.pdf]

Supplementary material has been published as submitted. It has not been copyedited, or typeset by Acta Oncologica

Supplementary table 1: Detailed overview of tumour types, treatment responses and mutational profile, all treated patients (n=65)

**Patient no.**	**Assay type**	**Cancer type**	**Radiological response after w16 (confirmed)**	**Gene alteration**	**Type of alteration**	**Variant**
Patient 01	TSO500	Acute myeloid leukemia	NE	KRAS	Activating mutation (missense)	Q61P
Patient 01	TSO500	Acute myeloid leukemia	NE	NRAS	Activating mutation (missense)	G12S
Patient 02	TSO500	Chronic myeloid neoplasia	Treatment stopped due to AE before W16	NRAS	Activating mutation (missense)	G12V
Patient 03	TSO500	Uveal melanoma	SD	GNAQ	Activating mutation (missense)	Q209P
Patient 04	TSO500	Colorectal carcinoma	PD before W16	NRAS	Activating mutation (missense)	Q61H
Patient 05	TSO500	Cholangiocarcinoma	PD before W16	NRAS	Amplification	CN >6
Patient 06	TSO500	Uveal melanoma	PD	GNAQ	Activating mutation (missense)	Q209L
Patient 07	TSO500	Glioblastoma	Treatment stopped due to AE before W16	NF1	Inactivating mutation (stop gain)	L2661X
Patient 07	TSO500	Glioblastoma	Treatment stopped due to AE before W16	NF1	Inactivating mutation (frameshift)	Y2285fs
Patient 08	TSO500	Low grade serous ovarian cancer	NE	KRAS	Activating mutation (missense)	G12V
Patient 09	TSO500	Uveal melanoma	SD	GNAQ	Activating mutation (missense)	G48L
Patient 10	TSO500	Lung adenocarcinoma	PD	NRAS	Activating mutation (missense)	Q61R
Patient 11	TSO500	Colorectal carcinoma	PD before W16	NRAS	Activating mutation (missense)	Q61K
Patient 12	TSO500	Glioblastoma	NE	NF1	Inactivating mutation (frameshift)	R1970fs
Patient 13	TSO500	Colorectal carcinoma	PD before W16	MAP2K4	Copy number loss	CN loss
Patient 14	TSO500	Uveal melanoma	SD	GNA11	Activating mutation (missense)	R183C
Patient 15	TSO500	Uveal melanoma	SD	GNA11	Activating mutation (missense)	Q209L
Patient 16	TSO500	Colorectal carcinoma	PD before W16	NRAS	Amplification	CN >6
Patient 17	TSO500	Neuroendocrine tumor, pancreas	PD before W16	BRAF	Activating fusion	SND1-BRAF
Patient 18	TSO500	Cholangiocarcinoma	SD	NRAS	Activating mutation (missense)	Q61R
Patient 19	TSO500	Glioblastoma	PD before W16	NF1	Inactivating mutation (frameshift)	C167fs
Patient 19	TSO500	Glioblastoma	PD before W16	NF1	Inactivating mutation (stop gain)	R2450X

Patient 20	TSO500	High-grade astrocytoma with piloid features	PD	NF1	Inactivating mutation (frameshift)	T2108fs
Patient 21	TSO500	Uveal melanoma	PD before W16	GNAQ	Activating mutation (missense)	Q209L
Patient 22	TSO500	Neuroendocrine tumor, pancreas	PD before W16	NRAS	Activating mutation (missense)	Q61K
Patient 23	TSO500	Uveal melanoma	PD	GNAQ	Activating mutation (missense)	Q209L
Patient 24	TSO500	Low grade serous ovarian cancer	SD	KRAS	Activating mutation (missense)	G12V
Patient 25	TSO500	Low grade serous ovarian cancer	Treatment stopped due to AE before W16	KRAS	Activating mutation (missense)	G12V
Patient 26	ctDNA	Uveal melanoma	SD	GNAQ	Activating mutation (missense)	Q209P
Patient 27	TSO500	Uveal melanoma	Treatment stopped due to AE before W16	GNA11	Activating mutation (missense)	Q209L
Patient 28	TSO500	Uveal melanoma	SD	GNA11	Activating mutation (missense)	Q209L
Patient 29	TSO500	Uveal melanoma	PD before W16	GNAQ	Activating mutation (missense)	Q209L
Patient 30	TSO500	Diffuse midline glioma, H3 K27-altered	NE	NF1	Inactivating mutation (stop gain)	K1977X
Patient 30	TSO500	Diffuse midline glioma, H3 K27-altered	NE	NF1	Inactivating mutation (frameshift)	Y2485fs
Patient 31	TSO500	Neuroendocrine carcinoma, cervix	PD before W16	NRAS	Activating mutation (missense)	G12D
Patient 32	TSO500	Lung adenocarcinoma	NE	NRAS	Activating mutation (missense)	Q61R
Patient 33	TSO500	Low grade serous ovarian cancer	SD	NRAS	Activating mutation (missense)	Q61R
Patient 34	TSO500	Ovarian cancer, fallopian tube	SD	KRAS	Activating mutation (missense)	G12V
Patient 35	TSO500	Uveal melanoma	PD	GNAQ	Activating mutation (missense)	Q209P
Patient 36	TSO500	Uveal melanoma	PD	GNAQ	Activating mutation (missense)	Q209L
Patient 37	TSO500	Cholangiocarcinoma	PD before W16	NRAS	Activating mutation (missense)	K117R
Patient 38	TSO500	Low grade serous ovarian cancer	PR	NRAS	Activating mutation (missense)	Q61R
Patient 39	TSO500	Mucosal melanoma	SD	BRAF	Activating fusion	MKRN1-BRAF
Patient 40	TSO500	High grade astrocytoma with piloid features	PR	NF1	Inactivating mutation (stop gain)	R1968X
Patient 41	TSO500	Uveal melanoma	PD before W16	GNA11	Activating mutation (missense)	Q209L
Patient 42	TSO500	Low grade serous ovarian cancer	PR	NRAS	Activating mutation (missense)	Q61R
Patient 43	TSO500	Low grade serous ovarian cancer	Withdrawn before W16	NRAS	Activating mutation (missense)	Q61R

Patient 44	TSO500	Glioblastoma	NE	NF1	Inactivating mutation (stop gain)	W2229X
Patient 45	TSO500	Endometrial carcinoma	NE	MAP2K4	Copy number loss	CN loss
Patient 46	TSO500	Low grade serous ovarian cancer	NE	KRAS	Activating mutation (missense)	G12D
Patient 47	TSO500	Ovarian cancer, mucinous adenocarcinoma	PD before W16	GNAS	Activating mutation (missense)	R201C
Patient 48	TSO500	Low grade serous ovarian cancer	SD	KRAS	Activating mutation (missense)	G12V
Patient 49	TSO500	Acute myeloid leukemia	PD	NRAS	Activating mutation (missense)	Q61K
Patient 50	TSO500	Diffuse leptomeningeal glioneural tumor	SD	BRAF	Activating fusion	KIAA1549-BRAF
Patient 51	TSO500	Low grade serous ovarian cancer	SD	KRAS	Activating mutation (missense)	G12V
Patient 52	TSO500	Recurrent pilocytic astrocytoma	SD	NF1	Copy number loss	CN loss
Patient 52	TSO500	Recurrent pilocytic astrocytoma	SD	NF1	Inactivating mutation (frameshift)	H122fs
Patient 53	TSO500	Neuroendocrine carcinoma, lung	PD before W16	HRAS	Activating mutation (missense)	Q61K
Patient 54	TSO500	Prostate cancer	NE	BRAF	Activating fusion	KIAA1549-BRAF
Patient 55	TSO500	Prostate cancer	PD before W16	MAP3K1	Copy number loss	CN loss
Patient 56	TSO500	Lung squamous cell carcinoma	NE	MAP2K4	Inactivating mutaton (missense)	c.514-1G>C
Patient 57	TSO500	Glioblastoma	PD	NF1	Copy number loss	CN loss
Patient 58	TSO500	Colorectal carcinoma	NE	NRAS	Activating mutation (missense)	A59T
Patient 59	TSO500	Low grade serous ovarian cancer	SD	KRAS	Activating mutation (missense)	G12D
Patient 60	TSO500	Low grade serous ovarian cancer	PR	KRAS	Activating mutation (missense)	G12D
Patient 61	TSO500	Pancreatic cancer	PD before W16	BRAF	Activating fusion	BRAF-TNS3
Patient 62	TSO500	Colorectal carcinoma	PD	NRAS	Activating mutation (missense)	Q61K
Patient 63	TSO500	Acute myeloid leukemia	NE	NRAS	Activating mutation (missense)	G12A
Patient 63	TSO500	Acute myeloid leukemia	NE	NRAS	Activating mutation (missense)	G13R
Patient 64	TSO500	Lung adenocarcinoma	NE	NRAS	Activating mutation (missense)	Q61L
Patient 65	TSO500	Colorectal carcinoma	PD before W16	NRAS	Activating mutation (missense)	Q61R

Supplementary table 2: Summarized overview of all treatment related adverse events according to Common Terminology Criteria for Adverse Events (CTCAE) v5.0, all treated patients (n=65). * SUSAR

**Adverse event CTCAE term**	**Grade 1**	**Grade 2**	**Grade 3**	**Grade 4**	**Grade 5**
Alanine aminotransferase increased			1		
Alkaline phosphatase increased			1		
Anorexia			1		
Arterial thromboembolism			1		
Aspartate aminotransferase increased			1		
CPK increased			2		
Colitis		1			
Cystitis noninfective			1		
Diarrhea	1	1	2		
Dyspnea		2			
Eczema		1			
Edema limbs		1			
Fatigue			3		
Febrile neutropenia			1		
GGT increased			1		
Gastric hemorrhage				1	
Heart failure			1		
Hypertension			1		
Hyponatremia			1		
Intra-abdominal hemorrhage					1
Intracranial hemorrhage			1		
Left ventricular systolic disfunction			1		
Lung infection			2		
Mucositis oral			4		
Myocardial infarction*			1		
Obstipation		1			
Pruritus			1		
Rash acneiform		1	5		
Rash maculo-papular			5		
Rash pustular			2		
Sepsis*			1		
Stroke*			1		
Thromboembolic event*			2		1
Urethral mucositis			1		
Vomiting			1		
